# Osteoclast Recycling and the Rebound Phenomenon Following Denosumab Discontinuation

**DOI:** 10.1007/s11914-022-00756-5

**Published:** 2022-10-06

**Authors:** Albert S. Kim, Christian M. Girgis, Michelle M. McDonald

**Affiliations:** 1 Bone Biology Program, Garvan Institute of Medical Research, Sydney, Australia; 2 Faculty of Medicine UNSW Sydney, St Vincent’s Clinical School, Kensington, NSW Australia; 3 Department of Diabetes and Endocrinology, Westmead Hospital, Westmead, NSW Australia; 4 Faculty of Medicine and Health, University of Sydney, Sydney, NSW Australia; 5 The Westmead Institute for Medical Research, Westmead, NSW Australia

**Keywords:** Osteoclast, Denosumab discontinuation, Osteoporosis

## Abstract

**Purpose of Review:**

Inhibition of receptor activator of nuclear factor kappa-B ligand (RANKL) with denosumab is an effective treatment in a number of conditions including osteoporosis where suppression of bone resorption is desired. However, denosumab discontinuation is associated with rebound increase in bone resorption and subsequent loss in bone mass and a rapid return to baseline fracture risk. We review recent data on the rebound increase in bone resorption following denosumab discontinuation and the potential mechanisms behind this phenomenon.

**Recent Findings:**

Osteoclasts have been considered to be highly specialised cells that undergo apoptosis after fulfilling their function of bone resorption. However, recent studies suggest that osteoclasts are longer lived cells which migrate through vasculature and are capable of undergoing fission into a novel cell type (the osteomorph) and re-fusion in a process termed osteoclast recycling.

**Summary:**

The life cycle of the osteoclast is more complex than previously appreciated. Osteoclast recycling provides a novel mechanistic framework to examine changes in osteoclast biology in response to treatment of bone diseases and provides an exciting new avenue towards personalised medicine.

## Introduction

The human skeleton is constantly turning over in a tightly regulated process known as bone remodelling. Bone remodelling occurs at a cellular level within a microscopic unit known as the basic multicellular unit (BMU), which consists of osteoblasts and osteoclasts coupled together on the bone surface, interacting through cell-to-cell contact and local and systemic cytokine networks, to regulate bone resorption and formation and maintain bone homeostasis [1]. Several bone diseases, such as osteoporosis, lead to an imbalance in bone remodelling resulting in net increase in bone resorption and subsequent bone loss. The osteoclast is the primary cell responsible for bone resorption and therefore has been the therapeutic target for several agents directed towards inhibiting bone resorption and increasing bone mass. Historically, osteoclasts have been thought to undergo apoptosis following completion of bone resorption. Alternative osteoclast cell fates such as fission had been postulated but were unable to be confirmed until recently. Advances in lineage tracing, single cell RNA sequencing and imaging technologies have challenged this long-held dogma. This review will briefly outline the historical life cycle of the osteoclast, from formation to fate. We will then focus on how recent discoveries have uncovered an increased life cycle and a novel cell fate, namely osteoclast recycling, and the relevance of this discovery to current and emerging therapies targeting bone resorption.

## Osteoclast Formation and Function

**Fig. 1:**
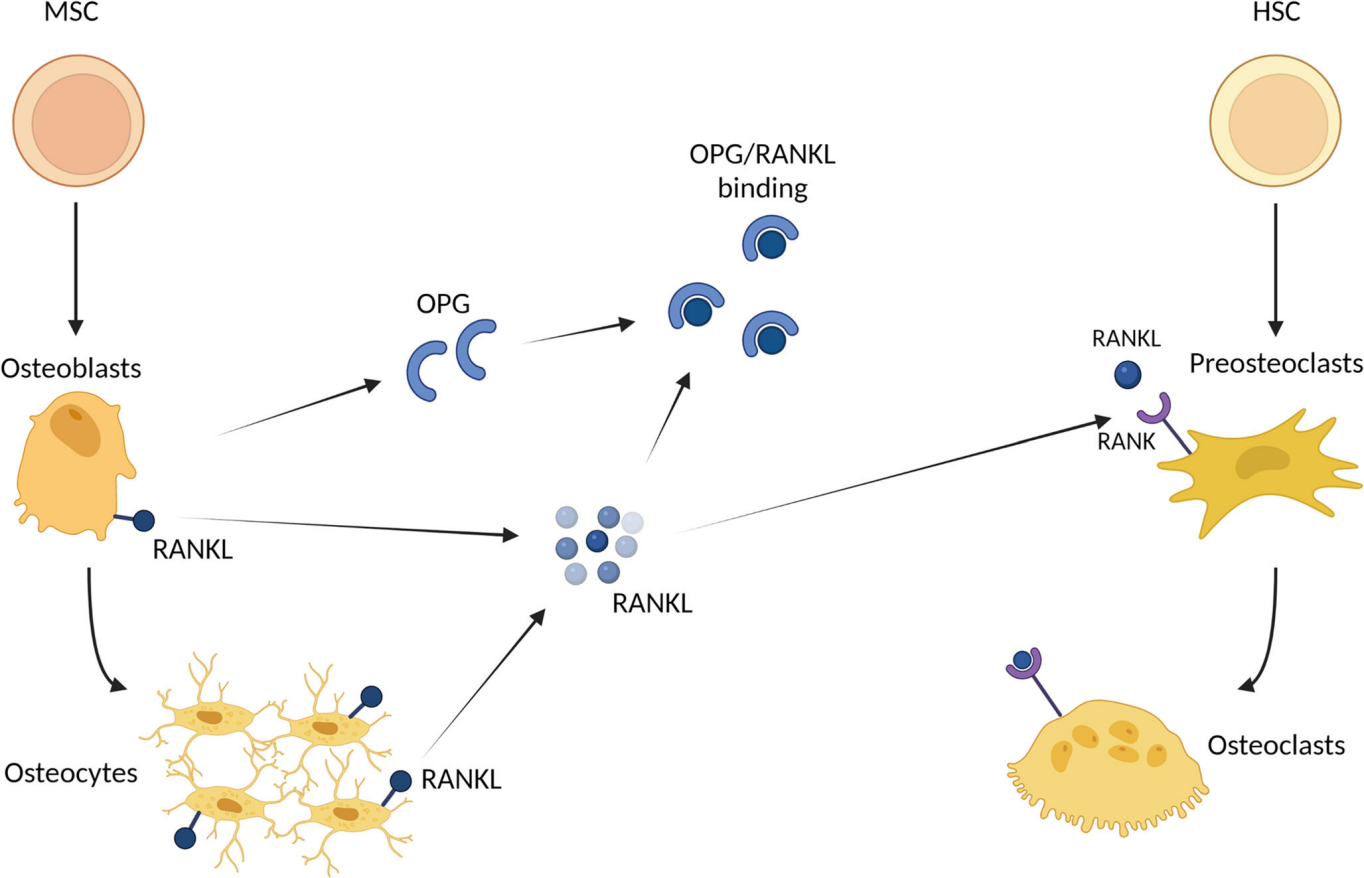
RANKL/RANK/OPG pathway. RANKL is produced by cells of the osteoblast lineage, including matrix-embedded osteocytes. Membrane bound RANKL is cleaved by proteases to form soluble RANKL. OPG is predominantly secreted by osteoblasts to bind to RANKL to suppress its activity and regulate osteoclastic bone resorption. RANKL binding to its receptor RANK promotes the differentiation of mature osteoclasts which are capable of attaching to and resorbing bone. Abbreviations: RANKL, receptor activator of nuclear factor kappa beta ligand, OPG, osteoprotegerin, MSC, mesenchymal stem cells, HSC, haematopoietic stem cells

The osteoclast is a highly specialised, multinucleated, tissue-specific macrophage responsible for bone resorption. Bone resorption by the active osteoclast occurs through the development of an acidic environment in which H+ ions are transported via proton pumps [2] and lytic enzymes tartrate resistant acid phosphatase (TRAP) and cathepsin K [3] are released into the resorption compartment. This dissolves hydroxyapatite and allows enzymatic degradation of the bone matrix proteins which are then phagocytosed by the osteoclasts and excreted [4]. Osteoclasts differentiate from monocyte precursors at or near the bone surface. Three molecules: receptor activator of nuclear factor kB ligand (RANKL) and its receptor RANK, and the decoy receptor osteoprotegerin (OPG), form the RANKL/RANK/OPG pathway (Fig. 1) which plays a critical role in the differentiation of osteoclasts from its haematopoietic progenitors.

### Osteoclastogenesis and the RANK/RANKL/OPG Pathway

Our current understanding of osteoclast differentiation and activity comes from initial observations made in animals and patients with osteopetrosis, a condition of increased bone mass due to arrested bone resorption. Pioneering parabiosis experiments in the 1970s showed restoration of bone resorption in osteopetrotic mice [5, 6], showing that precursors of these resorptive cells are of haematopoietic origin. Subsequent co-culture experiments [7] provided supporting evidence for a hypothesis that osteoblasts played a key role in mediating osteoclastogenesis [8], revealing that osteoclast formation requires physical contact between haematopoietic precursor cells with specific bone marrow derived stromal cells [9, 10]. This led to the discovery of OPG which plays a key role in osteoclastogenesis.

OPG is a protein of the TNF receptor superfamily and is predominantly secreted by osteoblasts and osteocytes [11]. Transgenic mice overexpressing the gene *Tnfrsf11b* encoding OPG displayed an osteopetrotic phenotype due to a profound decrease in osteoclasts [12]. Conversely, targeted ablation of OPG in mice led to severe osteoporosis due to marked increase in osteoclastogenesis and bone resorption [13]. Hence, OPG was determined a negative regulator of osteoclastogenesis.

OPG expression is regulated by Wnt signalling in osteoblasts [14] and local production of OPG by osteoblasts, rather than osteocytes. Interestingly, circulating OPG levels were unchanged in mice with conditional deletion of *Tndrsf11b* in osteoblasts which exhibited reduced cortical thickness and cancellous bone in the femur, demonstrating that local OPG is essential in regulating bone resorption [15]. OPG therefore was determined to be a decoy receptor for RANKL, blocking RANK and RANKL interaction and subsequent osteoclastogenesis.

RANKL is initially produced as an integral membrane bound protein but can be cleaved by proteases into a functional soluble form [17]. RANKL binds to its receptor RANK on osteoclast progenitors and stimulates osteoclast differentiation and function [16]. RANKL is involved in the fusion of osteoclast precursors into multinucleated cells, differentiation into mature osteoclasts and continued survival [17]. RANKL relies on macrophage-colony stimulating factor (M-CSF, also known as CSF-1) as a co-factor for osteoclast differentiation [18].

Genetic deletion studies show that RANKL is produced at various stages of the osteoblast lineage, including matrix-embedded osteocytes, though the relative contribution of RANKL at each stage remains unclear. Osteopetrosis was demonstrated in mice with RANKL deletion throughout the osteoblast lineage, and less so in mice with deletion restricted to differentiated osteoblasts and osteocytes only [19, 20]. Soluble RANKL, cleaved from its membrane bound form, is measurable in the circulation and increases with stimulated bone resorption [21]. Recent studies have demonstrated that the membrane-bound form of RANKL is responsible for the majority of RANKL functions and more potent than soluble RANKL in stimulating osteoclastogenesis in vitro [22]. This highlights the importance of the local microenvironment and cellular interactions between the different cell types within the BMU.

Elucidation of the RANK/RANKL/OPG pathway has revolutionised our understanding of osteoclastogenesis. Under the influence of RANKL and M-CSF and regulated by OPG, mononuclear haematopoietic cells of the monocyte lineage differentiate and fuse to form multinucleated osteoclasts capable of resorbing bone. However, until recently, little has been known about what happens to osteoclasts following completion of bone resorption.

## Osteoclast Cell Fate and Recycling

Following bone resorption, mature osteoclasts are typically thought to undergo apoptosis at the end of their lifespan of approximately 2–3 weeks, and hence, the number of osteoclasts is dependent on the rates of osteoclast differentiation and death [23]. This has been a long-standing dogma, initially described in 1920 where osteoclasts were no longer visible after bone resorption due to degeneration [24] and supported by observations of reduced osteoclast numbers following cessation of bone resorption [25, 26].

The first reported evidence of osteoclast apoptosis in vitro was in 1993 where mature osteoclasts died when the cultures were depleted of M-CSF. The authors concluded that the survival of mature osteoclasts occur through the suppression of apoptosis by factors such as M-CSF [27]. While RANKL and M-CSF are sufficient for osteoclast differentiation and enhance osteoclast survival, several proinflammatory cytokines, including TNFa, IL-1 and IL-6, have been shown to enhance osteoclast survival through activation of the NF-kB pathway [28, 29]. Parathyroid hormone (PTH) is an important hormone in calcium homeostasis as it stimulates osteoclast formation through RANKL expression by osteoblasts and reducing OPG expression by stromal cells [30, 31]. Therefore, proinflammatory cytokines and PTH predominantly act to inhibit osteoclast apoptosis by enhancing RANKL signalling to increase survival.

Conversely, several endogenous inducers of osteoclast apoptosis have also been identified which work through inhibition of RANKL signalling. In cultures, OPG enhances osteoclast apoptosis in a dose-dependent manner and upregulating molecular controllers of programmed cell death, including Fas ligand and caspase [32]. The important role of oestrogen in the regulation of bone homeostasis is evident in the pathophysiology of postmenopausal osteoporosis, where accelerated bone loss occurs with decreased circulating oestrogen in postmenopausal women [33]. Oestrogen appears to have a direct influence on osteoclasts with selective ablation of the oestrogen receptor on osteoclasts leading to trabecular bone loss in female mice and induction of apoptosis and upregulation of Fas ligand [34]. Oestrogen also indirectly promotes osteoclast apoptosis in vivo and in vitro which is mediated in part by transforming growth factor-B (TGF-B) signalling [35].

However, the role of these so called pro-apoptotic factors on osteoclast survival in vivo remains unclear. Indeed OPG has also been shown to suppress osteoclast apoptosis through the inhibition of TNF-related apoptosis inducing ligand (TRAIL) [36]. Oestrogen has been shown to reduce osteoclast numbers attached to bone and changes their morphology and size [37] which was accompanied by increased osteoclasts in the marrow space. This suggests that oestrogen treatment causes dissociation and morphological changes in osteoclasts rather than driving apoptosis, contrary to previous studies showing induction of apoptosis with ablation of the oestrogen receptor [34, 35].

Bisphosphonates are inhibitors of bone resorption and have revolutionised the treatment of a variety of bone diseases where excessive osteoclast activity is a pathological feature [38]. Bisphosphonates are selectively taken up and adsorbed to bone surfaces where they are internalised by osteoclasts, leading to loss of osteoclast function and induction of apoptosis. The mechanism via which bisphosphonates induce apoptosis differs between the two pharmacological classes of bisphosphonates. The nitrogen-containing bisphosphonates (such as alendronate and zoledronate) inhibit protein prenylation in osteoclasts [39], whereas non-nitrogen-containing bisphosphonates (such as clodronate) inhibit adenosine triphosphate (ATP)-dependent enzymes. This leads to osteoclast apoptosis [40], loss of the ruffled border and prevents attachment to bone [41]. Through these mechanisms, bisphosphonates increase bone mass and is now well established as an effective antiresorptive agent in the treatment of osteoporosis.

### Osteoclast Recycling

While osteoclasts have been thought to undergo apoptosis following bone resorption or under the influence of proapoptotic factors, several historic studies postulated an alternative cell fate. Osteoclast fission had been hypothesised following observations under microscopy, though this was not able to be confirmed [42]. Decreased number of nuclei per osteoclast followed by an increases in the number of osteoclasts was observed in the presence of calcitonin in vivo [25] and while this was thought to be due to fission of pre-existing osteoclasts, again this could not be confirmed. Advances in live cell imaging allowed the novel observation of osteoclast fission in vitro in 2012, where multinucleated osteoclasts were observed to split up into smaller, functional cells [43•].

More recent studies also challenge the long-held dogma that osteoclasts undergo apoptosis following bone resorption after a lifespan of 2–3 weeks and support an alternative cell fate of the osteoclast. Recent parabiosis and cell-fate studies have shown that osteoclasts are longer lived with a lifespan of around 6 months [44•, 45•]. Advances in single cell RNA sequencing and intravital imaging have revealed that osteoclasts behave as long-lived cells that circulate and undergo fusion and fission [46]. Mixed bone marrow chimeras were used to form osteoclasts that are formed through the fusion of bone marrow cells where the cells express a green or red fluorescent protein. The reporter proteins were driven by an osteoclast gene LysozymeM-tdTomato and CSF1R or Blimp-1-GFP leading to formation of multinucleated osteoclasts that express both tdTomato and GFP following cell fusion. These cells were multi-nucleated, secreted cathepsin K and were capable of resorbing fluorescently labelled bisphosphonate from bone, confirming their functional capacity as osteoclasts [47•].

Intravital imaging of these cells under RANKL stimulation revealed that osteoclasts undergo fusion in vivo and provided the first in vivo evidence of osteoclast fission. These fission events were distinct from osteoclast apoptosis. The fate of the fission products was tracked and were demonstrated to fuse with neighbouring osteoclasts and with each other, in a process now termed osteoclast recycling. These recycling cells, termed osteomorphs, are detectable in the blood and bone marrow and are identifiable as a unique cell population expressing 151 unique genes when compared to osteoclasts and osteoclast precursors using scRNA sequencing [47•].

Detailed skeletal phenotyping of mouse lines with single-gene deletions was available through the Origin of Bone and Cartilage Disease program [48], allowing the examination of 40 mouse lines in which one or both copies of an upregulated osteomorphs gene was deleted. A number of these genes were associated with skeletal phenotypes, suggesting that these osteomorphs genes pay a role in the regulation of skeletal structure and function. Analysis of human orthologs showed that osteomorph genes were strongly associated with changes in estimated bone mineral density (BMD) [47•].

**Fig. 2:**
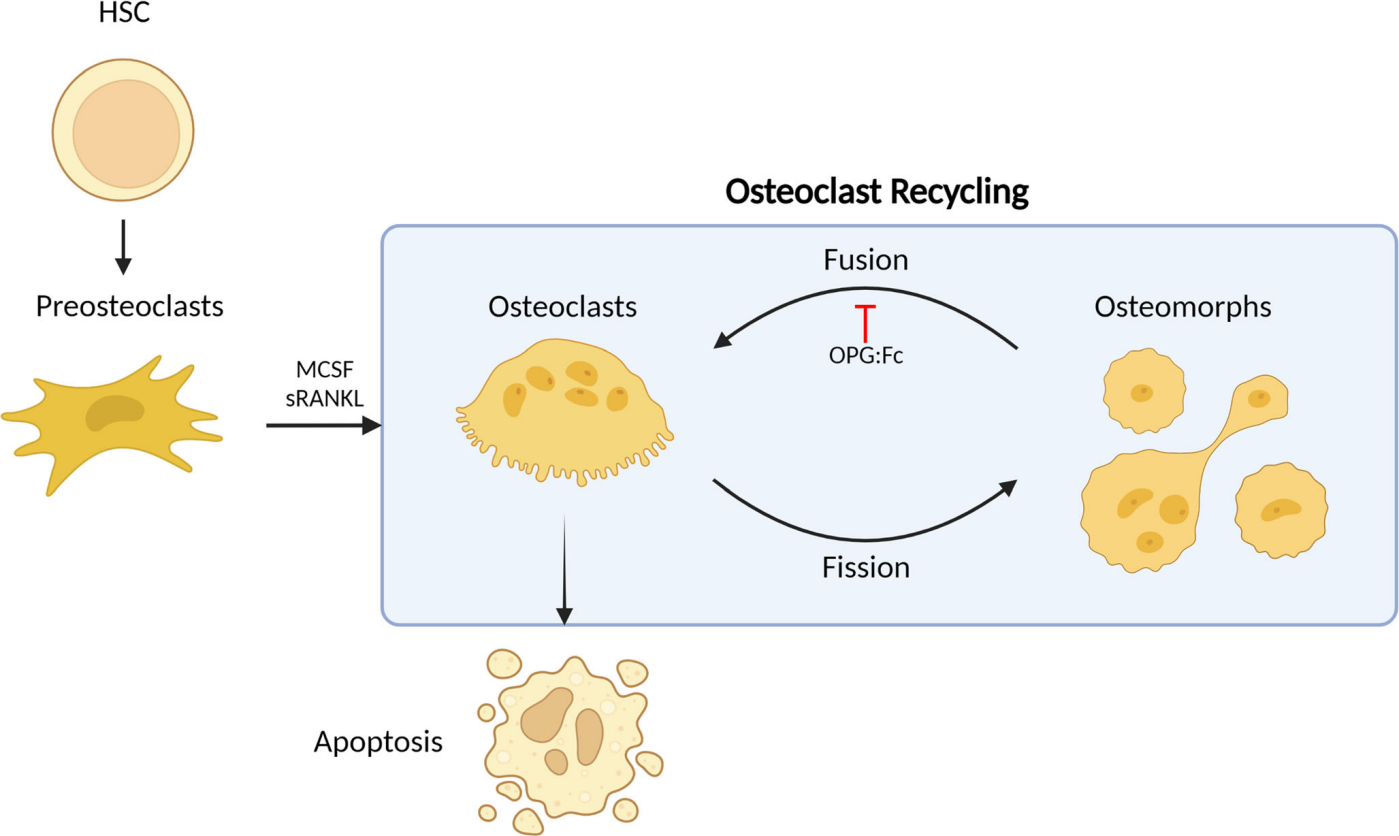
Osteoclast recycling. Osteoclasts have been thought to undergo apoptosis at the end of its life cycle. Under RANKL stimulation, osteoclasts are capable of undergoing fission into daughter cells termed osteomorphs which can circulate and fuse to re-form osteoclasts in a process termed osteoclast recycling. This is regulated by RANKL signalling and inhibition of RANKL with OPG:Fc leads to the accumulation of osteomorphs and osteoclast precursors (preosteoclasts). Abbreviations: RANKL, receptor activator of nuclear factor kappa beta ligand, OPG, osteoprotegerin, HSC, haematopoietic stem cells, MCSF, macrophage colony stimulating factor

Furthermore, inhibition of RANKL with OPG:Fc treatment led to the ablation of osteoclasts and the accumulation of osteoclast precursors and osteomorphs. These results indicate that osteoclast recycling is regulated by RANKL signalling and RANKL inhibition leads to the accumulation of osteomorphs which provides a pool of primed osteoclast precursors capable of re-fusing to form active osteoclasts when RANKL inhibition is discontinued (Fig. 2). This led to rapid resumption of bone resorption and reduced bone mass in these studies [47•]. Elucidating how osteoclast recycling is affected in different disease states could provide new avenues for personalised treatment of bone diseases [49]. In addition to challenging the long-standing dogma that osteoclasts have a linear fate ending in apoptosis, these discoveries also provide a mechanistic framework to examine emerging clinical phenomena observed with anti-RANKL therapies.

## Denosumab Discontinuation and the Rebound Phenomenon

### Denosumab

Inhibition of RANKL-RANK signalling leads to increased bone mass through inhibition of osteoclastic bone resorption. RANK- and RANKL-deficient mice display severe osteopetrosis due to arrested osteoclast differentiation [16, 50]. This led to the exploration of OPG as a therapeutic agent in the management of diseases where inhibition of osteoclastic bone resorption is desired. Although initial studies exploring the use of OPG-Fc showed promise in suppressing bone turnover in humans, further development was discontinued due to safety concerns over the development of neutralising immune response to endogenous OPG. Therefore, approaches targeting RANKL activity was preferred over increasing OPG, leading to the development of the anti-RANKL antibody denosumab [51].

Denosumab is a fully humanised monoclonal antibody that binds to both soluble and membrane bound RANKL with high affinity and specificity, thereby neutralising the effect of RANKL in a similar mechanism of action to OPG [52]. Denosumab reduces the number of osteoclasts and therefore increases bone mineral density [51]. Denosumab circulates in the bloodstream and is cleared through the reticuloendothelial system with a half-life of approximately 26 days. Unlike bisphosphonates, denosumab is not incorporated into the bone matrix.

Denosumab has revolutionised the management of osteoporosis. The efficacy of denosumab has been demonstrated in the landmark FREEDOM trials and has been associated with reduced fractures [53]. Treatment with denosumab leads to sustained increases in bone mineral density as long as treatment continues which was shown in a 10-year extension study [54]. This appears to be in part attributable to preservation of modelling-based bone formation during treatment [55].

Denosumab is a 6-monthy subcutaneous injection which is administered by a health professional. This is preferred and better tolerated by patients compared to bisphosphonate therapy [56]. This has led to progressive increases in denosumab prescription, and in Australia, denosumab is the most commonly prescribed antiresorptive agent [57]. Current guidelines recommend denosumab as first line therapy for the treatment of osteoporosis [58, 59]. Denosumab’s potent inhibition of osteoclast-mediated bone resorption has been utilised in other clinical settings where suppression of bone resorption is desired, often at higher doses and increased dose frequency. Adjuvant denosumab is used in patients with cancer to reduce the risk of clinical fractures related to cancer therapy [60, 61]. Denosumab is often utilised in paediatric bone diseases where increased BMD, reduced bone turnover and preventing growth of skeletal metastases is desired [62, 63].

### Rebound Phenomenon Following Denosumab Discontinuation

Patients receiving denosumab continue to experience BMD gains and fracture prevention. However, there are several clinical scenarios where denosumab discontinuation may be required. Drug “holidays” from antiresorptive therapy have been advocated to reduce the risk of rare, but serious, complications arising from long-term use such as atypical femoral fractures or osteonecrosis of the jaw [64, 65]. Adjuvant denosumab in cancer treatment may be discontinued when cancer treatment has completed. Patients may also develop contraindications to denosumab such as chronic kidney disease or need to consider a change in therapy due to inefficacy and ongoing fractures. Alternatively, patients discontinue denosumab as they are no longer at high risk of fractures or no longer have indications to continue treatment with denosumab. However, discontinuation of denosumab leads to rapid reversal of its therapeutic effect, leading to “rebound” bone loss to baseline bone density and fracture risk [66]. This was also observed in a post hoc analysis of the FREEDOM study cohort, showing an increase in vertebral fracture risk following denosumab discontinuation to the level observed in untreated participants [67].

Upon denosumab cessation and withdrawal of its effect, there is a rebound increase in bone resorption which is highlighted clinically as a rapid rise in the bone turnover markers. Both markers of bone formation, such as procollagen 1 intact N-terminal propeptide (P1NP), and bone resorption including C-terminal telopeptide (CTX) and TRAP-5b are increased following the offset of denosumab’s effect indicating a high bone turnover state [66, 68]. The net effect of this is bone loss, observed as a decrease in BMD which occurs throughout the skeleton and especially in the spine [66]. This has led to increases in the rates of preventable fractures following denosumab discontinuation [69]. Concerningly, prescription data in Australia shows that denosumab treatment is frequently discontinued or interrupted, placing many patients at increased risk of fractures [57].

**Fig. 3:**
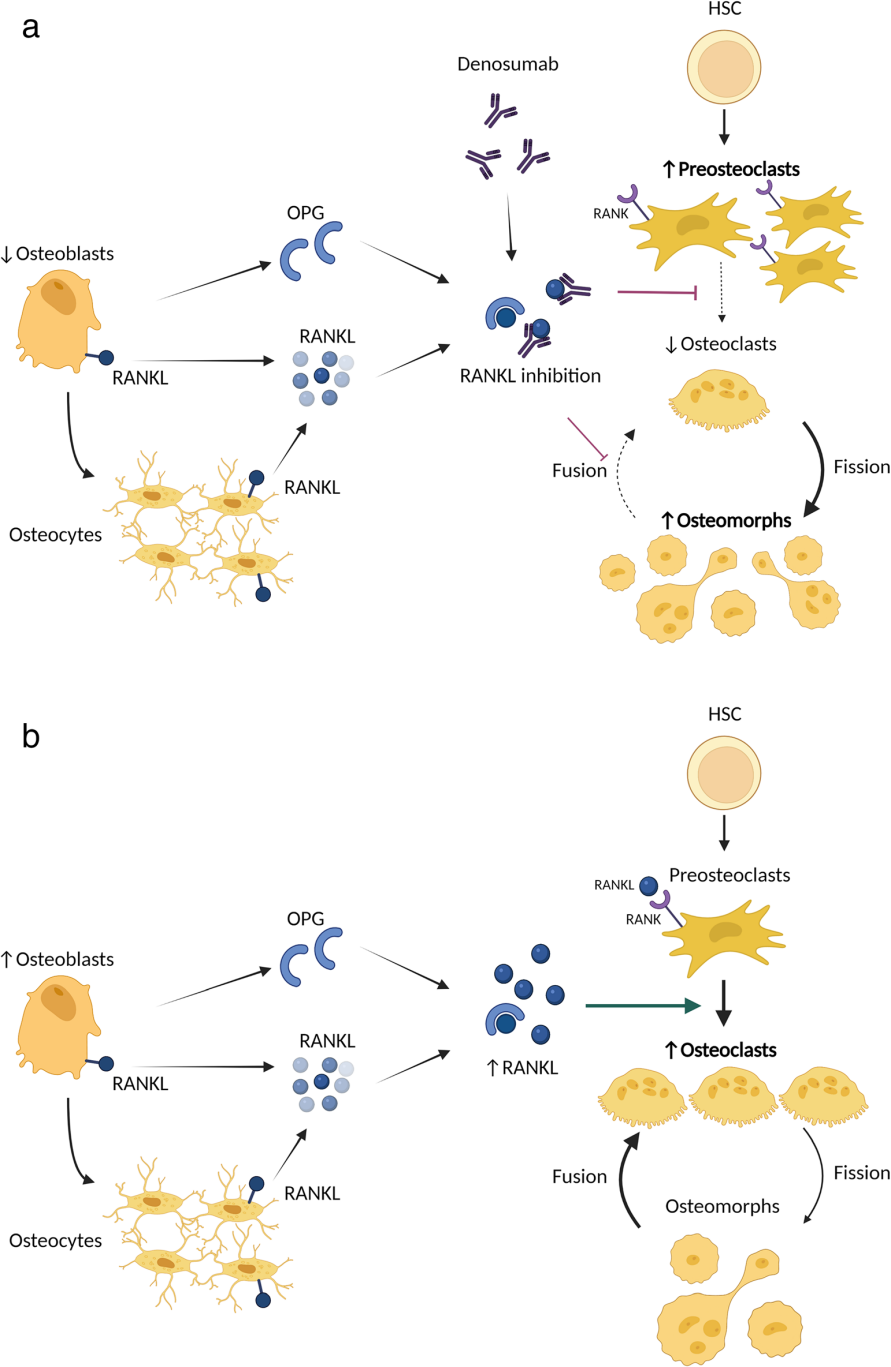
The effect of denosumab on osteoclast recycling. **A** Inhibition of RANKL with denosumab prevents the differentiation of preosteoclasts and leads to fission of osteoclasts into osteomorphs. Osteomorph fusion into osteoclasts is also inhibited by denosumab, leading to the accumulation of osteomorphs during denosumab treatment. **B** Denosumab discontinuation and its subsequent withdrawal of RANKL inhibition leads to increased RANKL and the resumption of osteoclast differentiation and osteomorphs fusion into osteoclasts. The accumulation of preosteoclasts and osteomorphs provide a pool of primed cells from which bone-resorbing osteoclasts can form. Abbreviations: RANKL, receptor activator of nuclear factor kappa beta ligand, OPG, osteoprotegerin, HSC, haematopoietic stem cells, MCSF, macrophage colony-stimulating factor

Significant increases in bone remodelling following denosumab discontinuation is accompanied by a rise in serum RANKL, though this only reached statistical significance after 12 months following the loss of effect of denosumab [70]. The mechanism underlying the delay in this RANKL rise is unclear but supports the possible mechanism where osteoclast precursors and osteomorphs form active, resorbing osteoclasts following the offset of denosumab effect (Fig. 3).

This is supported by an in vivo study examining the effect of mouse anti-RANKL monoclonal antibody which showed significantly increased TRAP-positive mononuclear cells in the bone marrow compared to controls in femoral sections following treatment discontinuation [71]. These mononuclear cells were predominantly found near, but not on, trabecular bone surfaces which may represent the fission of osteoclasts and the accumulation of osteoclast precursors and osteomorphs with anti-RANKL treatment. There was also increased expression of pro-osteoclastic genes including *C-fms*, *RANK* and *RANKL* in the bone marrow [71] suggesting a pro-osteoclastogenic bone marrow environment during RANKL inhibition. Thereby following treatment discontinuation, this leads to resumption of bone resorption and bone loss. This is consistent with the process of osteoclast recycling, where inhibition of RANKL with OPG:Fc led to the fission of osteoclasts into osteomorphs which then recycled to form resorbing osteoclasts once the RANKL inhibition was withdrawn.

Further evidence of this in humans is recent reports of accumulating of osteoclast precursors in the circulation of postmenopausal women receiving denosumab [72]. This supports the concept of a block in differentiation of osteoclast precursors and the fission of osteoclasts into osteomorphs during denosumab therapy. This may therefore lead to a pool of osteoclast precursors and osteomorphs primed to differentiate into osteoclasts and resorb bone once their inhibition by denosumab is withdrawn (Fig. 3).

Rebound increase in bone resorption following treatment discontinuation is not typically observed in patients treated with bisphosphonates. In ovariectomised (OVX) mice, risedronate demonstrated increased bone mass and suppression of bone turnover, whereas OVX mice that discontinued anti-RANKL antibody treatment experienced bone loss associated with an increase in bone turnover [73]. This may be due to the differences in the offset of antiresorptive activity between bisphosphonates, which are embedded into the bone matrix and therefore have a long duration of effect, compared to anti-RANKL antibodies which display a more rapid offset of effect as the drug is cleared from circulation. How bisphosphonates affect osteoclast recycling is yet unclear. Examination of osteoclast precursors in postmenopausal women treated with bisphosphonates showed a reduction of osteoclast precursors with treatment compared to healthy untreated controls, and there was no significant effect on RANKL and OPG levels [74]. As bisphosphonates inhibit bone resorption by predominantly acting on mature osteoclasts, it may not have a direct effect on osteoclast recycling. However, an apparent rebound increase in bone resorption following treatment discontinuation in OVX mice treated with NE-58025, a bisphosphonate with a low hydroxyapatite binding affinity, has been observed but the mechanism behind this remains unclear [75]. Studies to directly examine the effect of bisphosphonates on osteoclast recycling are therefore warranted.

Bone biopsies allow visualisation of histomorphometric changes that occur following denosumab discontinuation. Iliac crest bone biopsies from patients who experienced rebound fractures following denosumab discontinuation were compared to biopsies from patients receiving denosumab and treatment-naïve patients. This study demonstrated elevated bone turnover in patients discontinuing denosumab, with increased number of osteoclasts and eroded bone surface, as well as higher osteoblast numbers and osteoblast-covered bone surface [76]. Furthermore, there was a reduction in cortical and trabecular bone structure, with reduced cortical thickness and significantly lower trabecular bone volume, demonstrating compromised bone structure in patients following denosumab discontinuation [76]. In addition, there were alterations in osteocyte histomorphometry with a significant reduction in viable osteocytes with denosumab treatment which persisted at 12 months post-denosumab discontinuation, highlighting the accumulation of apoptotic osteocytes and retention of old bone during treatment due to suppressed bone turnover. These changes in osteocyte morphology following denosumab discontinuation highlight the complex cellular interaction between osteoclasts and the cells of the osteoblast lineage, particularly given the latter’s role in local RANKL and OPG production. This presents potential therapeutic opportunities to prevent the rebound phenomenon following withdrawal of RANKL inhibition.

### Sequential Therapy Following Denosumab Discontinuation

Current approaches following denosumab discontinuation aim to prevent the rebound changes in bone remodelling to avoid bone loss and reduce risk of fractures [68]. Randomised controlled trials of sequential bisphosphonate, which prevent mature osteoclasts from resorbing bone, have not succeeded in consistently prevent rebound bone loss following denosumab discontinuation [77]. Sequential treatment with the PTH-analogue teriparatide following denosumab discontinuation leads to accelerated BMD loss [78]. PTH mediates bone homeostasis in a coupled manner, affecting both bone formation and bone resorption. PTH signalling in osteoblasts and osteocytes increases the RANKL/OPG ratio which recruits osteoclast precursors and stimulates osteoclastogenesis [79]. Therefore, the observed changes in BMD with transition from denosumab to teriparatide may reflect an accumulation of osteoclast precursors and osteomorphs during denosumab treatment and the formation of active resorbing osteoclasts, accelerated in the presence of PTH.

Targeting osteoblasts with newer therapies such as the sclerostin inhibitor romosozumab provides an alternate therapeutic target in the setting of denosumab discontinuation. Local production of OPG by mature osteoblasts play a critical role in suppressing RANKL activity and osteoclastogenesis [15]. Transgenic mice treated with denosumab showed almost complete absent osteoclasts and osteoblasts on the bone surface and gene expression analysis showed a striking reduction in OPG mRNA expression [80]. These findings highlight that the lack of osteoblasts and the OPG produced by these cells may contribute to the rebound bone resorption following denosumab discontinuation. Therefore, increasing osteoblastogenesis could be an alternative strategy for sequential therapy.

The effect of prior treatment on treatment response to romosozumab was examined in a real-world observational study in Japan which showed significant attenuation of bone mineral density response in patients with prior denosumab use [81]. The cellular changes that occur with transition from denosumab to ROMO remains unclear but most likely involves changes in RANKL and OPG signalling given that sclerostin promotes osteoclastogenesis via a RANKL-dependent pathway [82]. There is also documented decrease in RANKL:OPG following anti-sclerostin treatment [83]. Given the complex cellular interaction at play, a multi-pronged approach targeting not only osteoclasts, but also cells of the osteoblastic lineage may prove to be the most effective in preventing the rebound phenomenon following denosumab discontinuation. Furthermore, investigation into how osteoclast recycling could be directly targeted and also how it is affected by anabolic therapies would contribute significant insight into the optimal sequential treatment approach in these patients.

## Conclusion

The discovery of osteoclast recycling provides a novel mechanistic framework to examine osteoclast biology and their role in skeletal diseases. Osteoclasts are long-lived cells with a complex life cycle and are capable of fission into osteomorphs, circulating and recycling to maintain bone homeostasis. Changes in osteoclast recycling, including the accumulation of the novel osteomorphs and their fusion to form mature osteoclasts, provides a mechanism underlying the rebound phenomenon following denosumab discontinuation and paves the way to a better understanding of the cellular responses to therapies targeting bone. As we improve our understanding of how other bone-targeted therapeutics impact osteoclast recycling, we will optimise sequential therapy approaches to prevent denosumab withdrawal induced bone loss. Finally, this paradigm shift in osteoclast biology will lead to more targeted and optimal treatment strategies in patients with skeletal diseases in which osteoclast recycling may be implicated.
